# Predictors of Cephalic Vaginal Delivery Following External Cephalic Version: An Eight-Year Single-Centre Study of 447 Cases

**DOI:** 10.1155/2017/3028398

**Published:** 2017-04-23

**Authors:** Natalie Kew, Jacobus DuPlessis, Domenic La Paglia, Katherine Williams

**Affiliations:** ^1^The Royal Women's Hospital, Parkville, VIC, Australia; ^2^Western Health, Melbourne, VIC, Australia

## Abstract

*Introduction.* Our study aims to investigate and evaluate (1) rates of success of ECV for breech presentation at term at the Royal Women's Hospital in comparison to international standards; (2) mode of delivery following ECV; (3) factors influencing success rates of ECV at the Royal Women's Hospital.* Methods.* An audit of all women who underwent ECV between the years 2007 and 2014 at the Royal Women's Hospital as public patients was completed. Data parameters were collected from paper and electronic patient files at the Women's Hospital. Data was collected to analyse the effect of the following parameters on ECV success and birth outcome: age, parity, gestational age, BMI, AFI, and tocolytic use. These parameters were analysed to determine their effect on ECV outcome and birth outcome.* Results.* The Women's Hospital, Melbourne, has an ECV success rate of approximately 37%. Of the patients undergoing ECV, 29% proceeded to normal vaginal delivery. Predictors of successful ECV included low BMI, multiparity, and AFI more than 16 (*P* < 0.05). The only predictor of cephalic vaginal delivery following ECV was multiparity. Negative predictors of cephalic delivery were low AFI and nulliparity.* Conclusions.* The success rate of ECV at the Women's Hospital, Melbourne, is in line with global standards.

## 1. Introduction and Hypothesis

External cephalic version (ECV) is a procedure in which the fetus is rotated from the breech to the cephalic position manually through manipulation of the mother's abdomen. This procedure is generally performed at or near term in order to improve the mother's chances of having a vaginal cephalic birth. Ultrasound and cardiotocography (CTG) are performed on the day of the procedure to ensure foetal wellbeing. An infusion of a tocolytic, such as terbutaline, is often used to decrease uterine tone and thus improve the success rate of the procedure. After 10–15 m of infusion a concomitant ultrasound is conducted and the ECV is performed. Following the procedure, success or lack of success is verified by ultrasound scan and CTG and clinical observations are taken at 30 minutes to ensure maternal and foetal wellbeing. At this point, anti-D is administered to rhesus negative women. Women are often offered a second attempt if ECV is initially unsuccessful [1].

Numerous studies, including Gottvall and Ginstman [2], demonstrate that the use of ECV decreases the risks associated with a breech vaginal delivery and the risks associated with a caesarean section, such as transient tachypnoea of the newborn, increased risk of haemorrhage, and increased risk of deep vein thrombosis following the procedure. Grootscholten et al. [3] have demonstrated that ECV is a safe procedure with few contraindications and few risks. Numerous national organisations, such as the Royal College of Obstetricians and Gynaecologists [1], Royal Australian and New Zealand College of Obstetrics and Gynaecology [4], and the American College of Obstetricians and Gynaecologists [5] recommend that all women with a breech presentation at 35–42 weeks of gestation should be offered an ECV. Hutton et al. [6] conducted an international multicentre randomised controlled trial studying the timing of ECV and concluded that early ECV (defined as 34-35 weeks) increases the likelihood of cephalic presentation at birth but does not reduce the rate of caesarean section and may increase the risk of preterm birth, whereas other studies including Gottvall and Ginstman [2] and Bogner et al. [7] have demonstrated that ECV at term reduces the risk of caesarean section.

The aim of this study was to (1) assess rates of success of ECV for breech presentation at term at the Royal Women's Hospital in comparison to international standards, (2) assess mode of delivery following ECV, and (3) assess factors influencing success rates of ECV at the Royal Women's Hospital. It was predicted that higher AFI (>10 cm), low BMI (<20 kg/m^2^), higher parity (>1), higher maternal and gestational age, and tocolytic use would be positive predictive factors on ECV success. It was predicted that low AFI, high BMI, nulliparity, younger maternal age, and tocolytic use would have a negative predictive value on ECV success. Hypotheses were also made about the positive effect that higher AFI, low BMI, higher parity, and higher maternal and gestational age would have on achieving a cephalic vaginal delivery birth outcome. It was predicted that low AFI, high BMI, nulliparity, and younger maternal age might have a negative predictive value on a cephalic vaginal delivery.

## 2. Methods

A literature review was performed prior to data collection. A search of MeSH of PubMed (electronic database) from 1966 to 2015 was carried out with the following parameters: “external cephalic version” and “premature birth” and “outcomes”. The MeSH term “moxibustion” was excluded as this was felt not relevant to the study and articles used were limited to human studies that were published in English. The Cochrane review on external cephalic version (ECV), published ahead of print on 1 April 2015, was accessed to support this literature review. This reviewed many of the above studies and synthesized the data of original studies in the form of a systematic review.

A grey literature search was also conducted. Articles used to inform the Royal Women's Hospital Clinical Practice Guideline, as well as the RANZCOG and international guidelines including the Cochrane Central Register of controlled trials, were found via a Google search and have been included in the analysis. Background reading was done on secondary data sources such as the Managing Obstetrics Emergencies and Trauma (MOET) or Advanced Life Support Obstetrics (ALSO) handbooks, as it was felt important as they show how primary data has been interpreted by organisations involved in teaching clinical care of obstetric patients. HosData, of the Department of Health Victoria, was contacted and a research request submitted. Research report was produced detailing which public hospitals have performed an ECV in Victoria. Data obtained from this literature search was used in the design of the study and in the selection of parameters being assessed.

Patients included in the study were recruited from the Pregnancy Day Care Clinic Bookings Diary and from the Health Records Department at the Women's Hospital. Inclusion criteria were women who have been coded for an ECV between the years 2007 and 2014. This study was a retrospective audit of all women undergoing ECV for breech presentation by specialist or trainee obstetricians. There were no specific exclusion criteria; however records of private patients were not accessible and limited data was collected regarding these patients. Data collection was limited to data available through paper and electronic records available at the Medical Records Department of the Royal Women's Hospital. A notable limitation of this study is a lack of inclusion of data for private patients; women who were private patients were included in the predictors for ECV success but were excluded from the birth outcome following ECV data analysis due to availability of data. Patients with missing data were included in this study.

Data was collected to analyse the effect of the following parameters on ECV success and birth outcome: age, parity, gestational age, body mass index (BMI), amniotic fluid index (AFI), placental position, initial presentation, and tocolytic use. These parameters were analysed to determine their effect on ECV outcome and birth mode following ECV outcome. Data analysis was performed using STATA 13.1 software and logistic regression was performed for the different parameters to determine their effect on birth outcome. Where odds ratios and comparisons have been calculated for the different parameters, the entire population of 447 cases was used as the control group.

Data was analysed to assess the impact of age, parity, gestational age at time of ECV, BMI, AFI, and tocolytic use on ECV success. These parameters were stratified and odds ratios of successful ECV were calculated for each of the stratified parameters. Confidence intervals have been calculated at 95% and results have been deemed statistically significant when *P* < 0.05. The same analyses have been performed assessing the effect of age, parity, gestational age at time of ECV, BMI, AFI, and tocolytic use on birth outcome. These stratified parameters have been analysed for their predictive effect on cephalic vaginal delivery in patients undergoing ECV for breech presentation.

## 3. Results

A total number of 447 cases of women undergoing ECV for breech presentation were analysed in this study. This reflects approximately one-third of breech presentations that would be expected over the study time period, a figure which is calculated based on the assumption that breech presentations constitute 3% of all births. The results were calculated with binary outcomes for the different parameters analysed. The mean age of the women in the study was 31.8 years, which was consistent with Australia's median of 31 years for pregnant women [8]. The mean gravidity was 2 pregnancies, the mean parity of the women included in the study was 0.55, and the mean gestation at time of ECV was 37.3 weeks. The mean BMI was 24.1, and mean AFI was 11.6. No women were identified as Aboriginal or Torres Strait Islander, which could reflect a lower rate of Indigenous women accessing ECV. Demographic data is summarised in Table 1.

Analysis of the data revealed that statistically significant predictors of ECV success were high AFI, low BMI, and multiparity. There were no statistically significant findings for either gestation, age, or tocolytic use on ECV success. This is consistent with other medical studies on ECV predictors internationally and in Australia (Cho et al. 2012), [9]. Low AFI was found to be a statistically significant negative predictor of ECV. No other parameters studied were found to be statistically significant negative predictors of ECV success. The mode of delivery following successful ECV was most likely to be cephalic vaginal delivery, whereas the mode of delivery following failed breech tended to be an elective caesarean section. Data surrounding ECV success are summarised in Table 2.

Multiparity was found to be a significant predictor of ECV converting successfully to cephalic vaginal delivery. Age, BMI, gestation, AFI, and tocolytic use were not found to have a statistically significant relationship with ECV successfully converting to cephalic vaginal delivery. Nulliparity and low AFI were found to be negative predictive factors for conversion to cephalic vaginal delivery. Age, BMI, gestation, and tocolytic use were not found to have a negative effect on successful conversion to cephalic vaginal delivery. Data regarding predictors of cephalic vaginal delivery is summarised in Table 3.

In the analysis of the effect of age on both ECV success and successful conversion to cephalic vaginal delivery, there were no significant differences for any of these groups on either ECV success or successful conversion to cephalic vaginal delivery. It was hypothesised that higher gestational age at time of ECV would be a positive predictive factor for both ECV success and cephalic vaginal delivery following ECV. On analysis of data, no statistically significant results were found.

As hypothesised, low BMI was found to be a positive predictor of a successful ECV; the odds ratio for ECV success for women with a BMI of 15–20 was 1.71 (95% confidence interval 1.00 to 2.91, *P* < 0.05). Low BMI was found to be associated with normal cephalic delivery following ECV (OR 1.25); however, this result was not statistically significant. No statistically significant differences in rates of ECV success or rates of normal vaginal delivery were found in patient with a normal BMI. It was predicted in the study hypothesis that high BMI would have a lower chance of ECV success. This was not the case; being overweight (BMI 25–30) and obese with a BMI (BMI > 30) was not found to be a negative predictor of either ECV success or normal vagnial delivery. No statistically significant results were found for BMI > 20 on either ECV success or chance of cephalic vaginal delivery following ECV.

**Figure 1:**
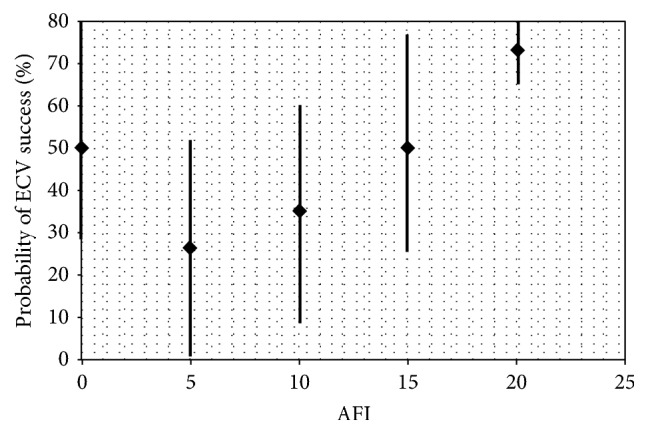
AFI versus probability of ECV success.

Low AFI was associated with ECV failure. An AFI of less than 10 was found to have an odds ratio of 0.63 (95% confidence interval 0.41 to 0.97, *P* < 0.05) as a predictor of ECV success. Similarly, as hypothesised, women with a high AFI were found to be more likely to have a successful ECV than the general cohort. An AFI of greater than or equal to 16 was found to be a strong predictor of success, with an odds ratio of 2.08 (95% confidence interval 1.26 to 3.45, *P* < 0.05). The relationship between AFI and ECV success is depicted in Figure 1. Similarly, as hypothesised, low AFI was found to be a statistically significant negative predictor of cephalic vaginal delivery following ECV (OR 0.53, 95% confidence interval 0.31 to 0.91, *P* < 0.05).

**Figure 2:**
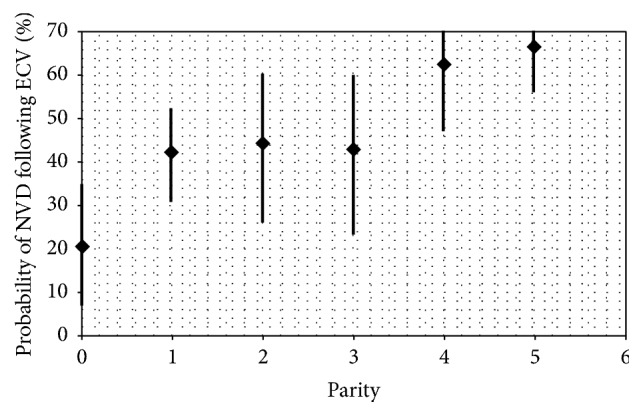
Parity versus probability of cephalic vaginal delivery following ECV. Diamond denotes calculated probability of cephalic vaginal delivery for a given parity.

Parity defined as a previous pregnancy past 20 weeks of gestation. This study found that, as hypothesised, multiparity was a statistically significant predictor of ECV success (OR of 1.54, 95% confidence interval 1.04 to 2.27, *P* < 0.05). It was hypothesised that nulliparity would be a negative predictor of ECV success and while the results indicate that this is the case, this result was not found to be statistically significant. Meanwhile, multiparity was also hypothesised to be a predictor of successful conversion to cephalic vaginal delivery. This was found to be the case, with an odds ratio of 1.91 (95% confidence interval 1.27 to 2.92, *P* < 0.05). Furthermore, nulliparity was also found to be a statistically significant predictor of failure to convert to cephalic vaginal delivery (odds ratio 0.67, 95% confidence interval 0.47 to 0.97). This relationship between parity and cephalic vaginal delivery is depicted in Figure 2.

Tocolytic use was hypothesised to be a predictor of both ECV success and conversion to successful cephalic vaginal delivery. This was not found to be the case; this study failed to yield any statistically significant results for the parameter of tocolytic use. This is contradictory to established literature (Cho 2012). It should be noted, however, that often when reviewing paper files there was no documentation for the parameter of tocolytic use; in the case of tocolytic use not being documented it was assumed that tocolytics were not used during ECV. It is likely that a proportion of these cases did indeed have tocolytics used, which would have influenced the data and may have underestimated the effect of tocolytic use.

Data was collected for birth outcomes following ECV. Data existed for birth outcome for 400 of the women in this study. Of these, 202 (50.5%) went for a planned caesarean section, 112 (28%) went for a cephalic vaginal delivery, 43 (10.75%) went for an emergency caesarean section for a medical indication, and 30 (7.5%) had an emergency caesarean section for breech presentation. Only three of these cases of emergency caesarean section were at the time of ECV for foetal bradycardia.

## 4. Discussion

Rate of success of ECV for breech presentation at term at the Royal Women's Hospital was 37%. Mode of delivery following successful ECV was normal vaginal delivery, whereas planed caesarean section was most likely following failed ECV. It is recommended that all women at term with a breech presentation be offered ECV in order to minimise the risk of noncephalic presentation at term and to reduce the risk of caesarean section [2]. The aim is to increase the chance of cephalic vaginal delivery at term. The overall success rate of ECV has been observed at approximately 50%. For every three ECVs performed, it has been shown that one caesaean section can be avoided [10].

As well as having a high chance of success, ECV is a safe procedure. Discomfort to the mother produced by the abdominal pressure performed during the procedure is an issue. This can be helped by tocolytic use, which increases success but carries a small risk of complications. The discomfort risk and tocolytic use risk is weighed up against the risks of the two management options available to the woman with a noncephalic fetus. Of the women studied, only 3 cases required an emergency caesarean section for foetal bradycardia during ECV and of these cases, neither the mother nor the baby had any adverse outcomes. Only three serious complications were reported, representing a complication rate of less than 1%, with three cases requiring an emergency caesarean section for foetal bradycardia and none of these cases had a documented adverse outcome for the mother or baby.

Previous studies have found that women with a higher BMI were less likely to have a cephalic vaginal delivery following an external cephalic version [11]. In the analysis of data from women at the Royal Women's Hospital, BMI was stratified into four groups: 15 to 20, 20.1 to 25, 25.1 to 30, and greater than or equal to 30.1. None of these groups were found to have an association with cephalic vaginal delivery. Only low BMI was found to be a predictor of ECV success.

The results of this study help predict the chance of a cephalic vaginal delivery following ECV. This information is useful when consenting women for ECV, where knowledge of the predictors of ECV success and of cephalic vaginal delivery can be used to help gain informed consent and to counsel women regarding the procedure.

In addition to these findings, there were several incidental findings in the study. Foetal abnormalities were found to be common in the studied cohort. In the data collection 8 cases of foetal anomalies were incidentally noted, and of 400 known birth outcomes this gives a congenital anomaly rate of 1 in 50. In this cohort there were two cases of tetralogy of fallot, or 0.5%, compared to the incidence of tetralogy of fallot in the general population of 1 in 2518 [12]. This is something to be noted in the management of breech presentations as well as planning future research study design. Furthermore, this information may be further studied to analyse the relationship between foetal presentation and foetal abnormalities.

## 5. Conclusions

The population in this study had an overall ECV success rate of 37%. Only three serious complications were reported during ECV, with three cases requiring am emergency caesarean section for foetal bradycardia and of these no babies had adverse outcomes. The mode of delivery following successful ECV was most likely to be cephalic vaginal delivery, whereas the mode of delivery following failed breech tended to be an elective caesarean section. Predictors of ECV success were higher AFI, low BMI, and multiparity. The only statistically significant predictor of cephalic vaginal delivery following a successful ECV was multiparity.

## Figures and Tables

**Table 1:** Demographic data.

	Mean	Standard deviation	Minimum	Maximum
Age (years)	31.8	4.7	17	43
BMI (kg/m^2^)	24.1	5.37	15	54.3
Gestation (weeks)	37.3	1.06	31	41
AFI (cm)	66	116	0.97	0.68 to 1.39
Parity (number)	0.55 (mode 1)	1.06	0	6

**Table 2:** Predictors of ECV success.

	Successful ECV (*n*)	Unsuccessful ECV (*n*)	OR	CI	*P*
Age (years)					
≤25	16 (47%)	18 (53%)	1.52	0.75 to 3.06	*P* = 0.24
26–30	30 (33%)	60 (67%)	0.85	0.53 to 1.38	*P* = 0.52
31–35	66 (36%)	116 (64%)	0.97	0.68 to 1.39	*P* = 0.88
>35	51 (39%)	79 (61%)	1.10	0.79 to 1.65	*P* = 0.63
Gestation (weeks)					
≤36	45 (37%)	78 (63%)	0.99	0.65to 1.49	*P* = 0.95
37	67 (34%)	131 (66%)	0.87	0.62 to 1.24	*P* = 0.45
≥38	44 (41%)	64 (59%)	1.18	0.76 to 1.80	*P* = 0.46
BMI (kg/m^2^)					
15–20	31 (50%)	31 (50%)	1.71	1.00 to 2.91	**P** = 0.049
20.1–25	60 (34%)	117 (68%)	0.88	0.61 to 1.26	*P* = 0.48
25.1–30	30 (37%)	51 (63%)	1.01	0.62 to 1.64	*P* = 0.98
≥30.1	13 (32%)	28 (68%)	0.79	0.40 to 1.57	*P* = 0.51
AFI (cm)					
≤10	36 (27%)	97 (73%)	0.63	0.41 to 0.97	**P** = 0.04
11 to 15	65 (35%)	119 (65%)	0.93	0.65 to 1.34	*P* = 0.71
≥16	39 (55%)	32 (45%)	2.08	1.26 to 3.45	**P** = 0.0044
Parity (number)					
0	92 (32%)	197 (68%)	0.80	0.58 to 1.09	*P* = 0.16
≥1	64 (47%)	71 (53%)	1.54	1.04 to 2.27	**P** = 0.03
Tocolytic use					
Yes	114 (39%)	178 (61%)	1.09	0.81 to 1.48	*P* = 0.56
No	47 (32%)	101 (68%)	0.80	0.54 to 1.18	*P* = 0.26

**Table 3:** Predictors of cephalic vaginal delivery in patients undergoing ECV.

	Cephalic vaginal delivery (*n*)	Other birth outcomes (*n*)	OR	CI	*P*
Age (years)					
≤25	12 (35%)	22(65%)	1.38	0.66 to 2.89	*P* = 0.39
26–30	21 (24%)	68 (76%)	0.78	0.46 to 1.34	*P* = 0.37
31–35	41 (29%)	102 (71%)	1.02	0.66 to 1.55	*P* = 0.94
>35	36 (32%)	76 (68%)	1.20	0.76 to 1.88	*P* = 0.44
Gestation (weeks)					
≤36	29 (27%)	79 (73%)	0.94	0.59 to 1.52	*P* = 0.81
37	45 (26%)	130 (74%)	0.89	0.59 to 1.33	*P* = 0.57
≥38	29 (30%)	69 (70%)	1.08	0.67 to 1.76	*P* = 0.75
BMI (kg/m^2^)					
15–20	17 (33%)	35 (67%)	1.25	0.67 to 2.32	*P* = 0.48
20.1–25	46 (26%)	131 (74%)	0.90	0.60 to 1.35	*P* = 0.62
25.1–30	21 (25%)	63 (75%)	0.86	0.50 to 1.47	*P* = 0.58
≥30.1	11 (29%)	27 (71%)	1.05	0.50 to 2.18	*P* = 0.9012
AFI (cm)					
≤10	19 (17%)	92 (83%)	0.53	0.31 to 0.91	**P** = 0.022
11 to 15	51 (30%)	118 (70%)	1.11	0.75 to 1.64	*P* = 0.60
≥16	20 (36%)	36 (64%)	1.43	0.79 to 2.57	*P* = 0.23
Parity (number)					
0	55 (21%)	210 (79%)	0.67	0.47 to 0.97	**P** = 0.036
≥1	54 (43%)	72 (57%)	1.92	1.27 to 2.92	**P** = 0.0019
Tocolytic use					
Yes	64 (26%)	184 (74%)	0.89	0.62 to 1.28	*P* = 0.54
No	44 (31%)	99 (69%)	1.14	0.75 to 1.73	*P* = 0.53

## Abbreviations

AFI: Amniotic fluid index
BMI: Body mass index
CVD: Cephalic vaginal delivery
ECV: External cephalic version
PDCC: Pregnancy Day Care Centre.
